# P-2140. Dalbavancin therapy failure in patients with Staphylococcus aureus infections despite sensitive MIC to Vancomycin: A retrospective Cohort

**DOI:** 10.1093/ofid/ofaf695.2303

**Published:** 2026-01-11

**Authors:** Kendymill Taveras, Margaret Cook, Stacy Harmon, Madeline Nowakowski, Mitchell Sorenson, Kans Lewis, Laura Mallinger, Megan Elmes, Rodolfo Alpizar-Rivas

**Affiliations:** Advocate Christ Medical Center, Orland Park, IL; Aurora St Luke's Medical Center, milwaukee, Wisconsin; Aurora St Luke's Medical Center, milwaukee, Wisconsin; Aurora St Luke's Medical Center, milwaukee, Wisconsin; Aurora St Luke's Medical Center, milwaukee, Wisconsin; Aurora St Luke's Medical Center, milwaukee, Wisconsin; Advocate Christ Medical Center, Orland Park, IL; Advocate Christ Medical Center, Orland Park, IL; Aurora St Luke's Medical Center, milwaukee, Wisconsin

## Abstract

**Background:**

Dalbavancin is a lipoglycopeptide antibiotic active against aerobic gram-positive organisms. It is approved to treat acute bacterial skin and skin structure infections (ABSSSI) with increasing interest in off-label use for blood stream infections (BSI), and osteomyelitis (OM). Its major benefit involves a long half-life. However, most clinical microbiology laboratories are unable to verify in vitro activity, resulting in limited real-time susceptibility testing. Therefore, the use of surrogate testing with susceptibility to other glycopeptide antibiotics test is used. This retrospective cohort evaluated the characteristics of treatment failures and re-occurrence of Staphlococcus aureus infections despite susceptibility to vancomycin.

**Figure:**
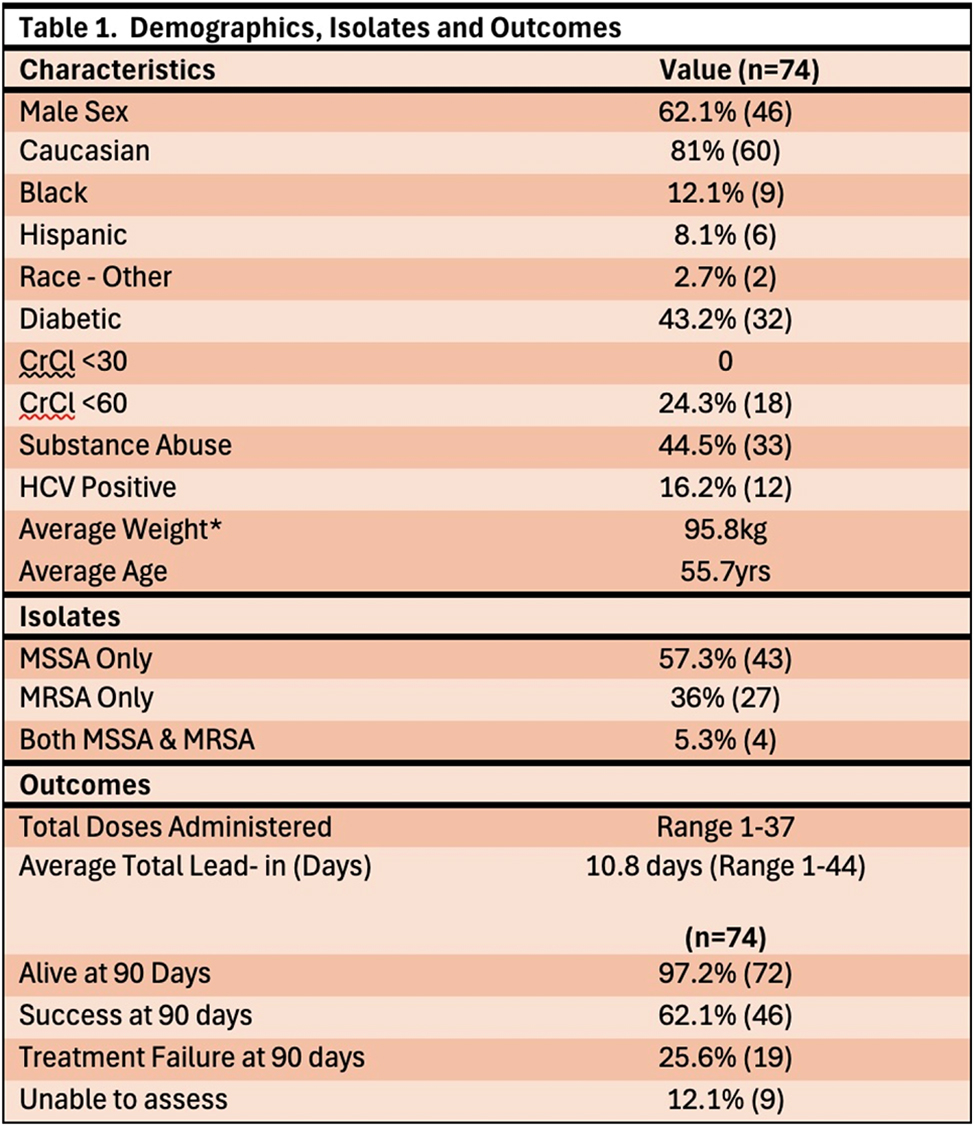

**Figure:**
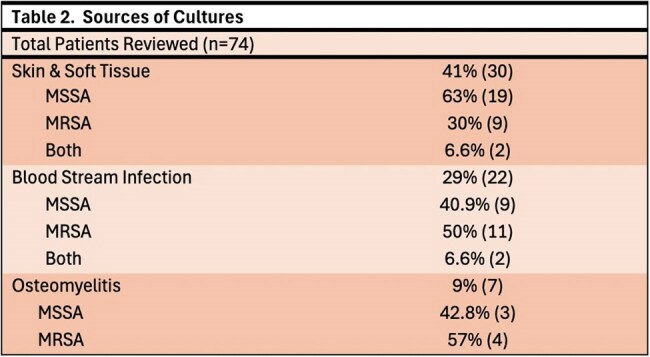

**Methods:**

We retrospectively identified 203 cases between 2023-2024 . We extracted baseline characteristics of patients with S. aureus infections. Patient age, gender, race, comorbidities, source of infection, vancomycin MIC, doses of dalbavancin and if companion antibiotics were used, were obtained. Treatment outcome was determined based on emergency department visits and hospital readmissions at 30-, 60-, and 90-days post-treatment.

**Figure:**
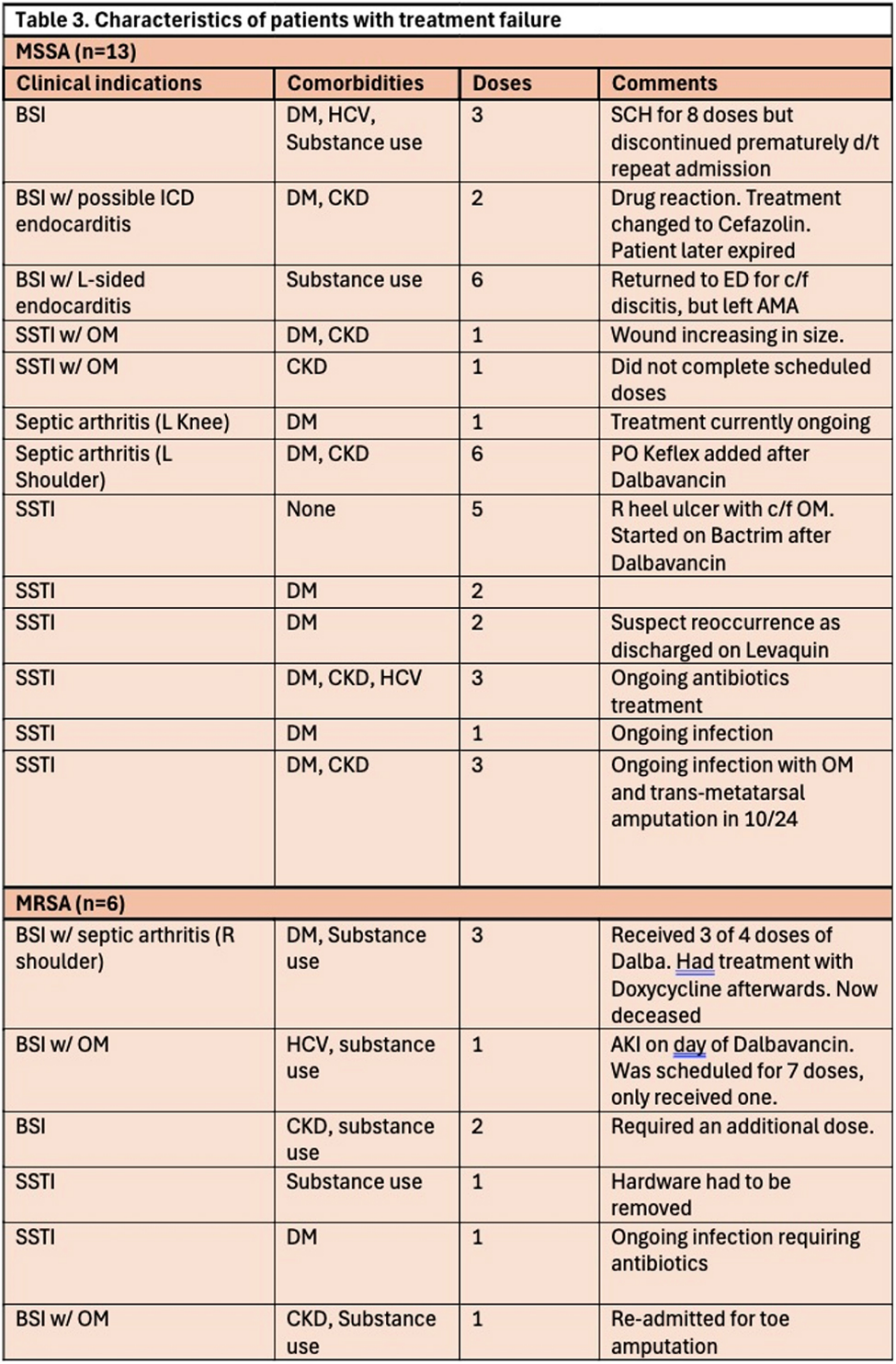

**Figure:**
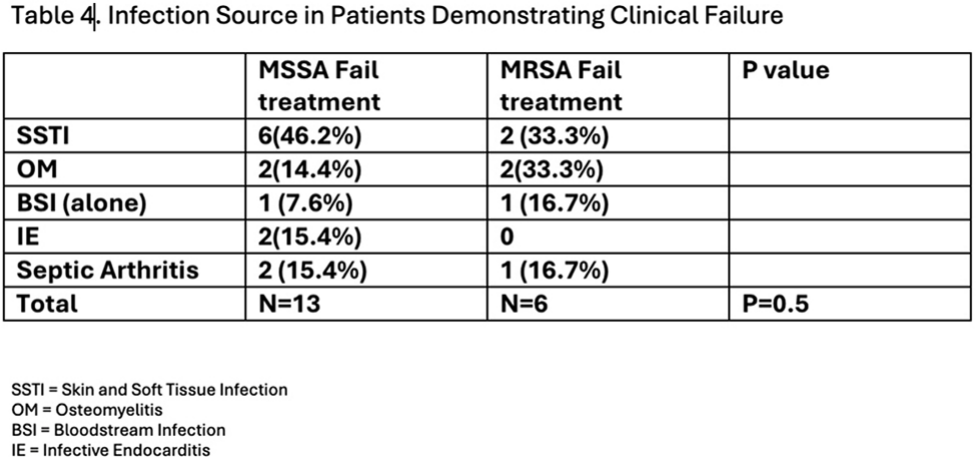

**Results:**

Of the 203 cases evaluated, 74 cases of S. aureus infections were isolated, with methicillin-sensitive S. aureus (MSSA) (n=43, 57.3%) and methicillin-resistant S. aureus (MRSA) (n=27, 36%). The most common clinical indications for dalbavancin use were ABSSSI and BSI at 41% and 29% respectively. In this cohort, 46 patients cleared the infection, 19 had treatment failure despite all showing vancomycin MIC < 0.5, and 9 were lost to follow up. Of the 19 failed treatments, 13 were due to MSSA infections and 6 were related to MRSA. The sources of infections in these treatment failures are detailed in Table 4. A subgroup analysis of treatment failures found a non-significantly higher failure rate noted in the MSSA group when compared to MRSA (p=0.5).

**Conclusion:**

This review found a non-significantly higher rate of treatment failure when dalbavancin was used for MSSA despite glycopeptide susceptibility. We suspect the difference was not statistically significant due to underpower. This review suggests further studies are warranted to examine dalbavancin off-label use in Staphylococcal infections.

**Disclosures:**

All Authors: No reported disclosures

